# Research note: Shedding a light on dustbathing preferences - Do hens prefer bright or dark dustbathing areas?

**DOI:** 10.1016/j.psj.2026.106988

**Published:** 2026-04-21

**Authors:** Catharina M.H. Broekmeulen, Thea G.C.M. van Niekerk, Arjan van Dolderen, Rudi M. de Mol, Dennis E. te Beest, Ingrid C. de Jong

**Affiliations:** a Animal Health and Welfare Department, Wageningen Livestock Research, Wageningen University and Research, P.O. Box 338, 6700 AH Wageningen, The Netherlands; b Biometris, Wageningen University and Research, P.O. Box 16, 6700 AA Wageningen, The Netherlands

**Keywords:** Preference test, Dustbathing, Substrate, Light intensity, Laying hen

## Abstract

Light intensity is an important factor in designing functional areas in poultry housing, as it influences behavioral activity. However, its influence on dustbathing behavior remains unclear. This study investigated whether laying hens prefer to dustbathe in brighter or darker areas, in combination with either a dark or light colored dustbathing substrate (i.e., peat or sand), using a preference test. Twenty-four hens, housed in pairs, were tested in three successive rounds (n = 8/ round) during which they were given a choice out of four different compartments containing sand at 5 lux, peat at 5 lux, sand at 100 lux, and peat at 100 lux. Dustbathing behavior and duration were recorded via video, and visits to the compartments were registered by RFID. Hens showed a clear preference for dustbathing in peat at 100 lux, as they performed more dust baths in this combination compared to sand at 100 lux, peat at 5 lux, and sand at 5 lux (P = 0.003, P = 0.002, P < 0.001, respectively). Hens visited the compartments with sand at 100 lux more often than those with sand at 5 lux (P = 0.02), while no other differences in visit frequency were found. Visit frequency substantially exceeded dustbathing events, suggesting that compartment visits may also reflect the motivation to explore or forage. Our findings provide a first indication that substrate preference and light intensity may interact to reinforce dustbathing behavior, which provides practical guidance for designing dustbathing areas in commercial housing systems.

## Introduction

Chickens perform dustbathing behavior to maintain plumage condition by removing excess and degraded lipids from the skin and feathers (Van Liere, 1991). In line with this functional role, laying hens generally prefer fine substrates such as sand or peat for dustbathing, although these preferences may be influenced by prior experiences (Van Liere, 1991; Sanotra et al., 1995; De Jong et al., 2007). However, substrate preference alone does not account for dustbathing behavior, as dustbathing motivation is also socially facilitated and appears to be intrinsically driven. Accordingly, hens reared with and without access to a dustbathing substrate are equally motivated to dustbathe (Duncan et al., 1998; Wichman and Keeling, 2008). To meet this behavioral need, commercial housing systems must provide appropriate dustbathing substrates (i.e., type, quality, quantity) as well as sufficient space to allow chickens to synchronize their dust baths (Van Liere, 1991; De Jong et al., 2007). In addition, environmental factors such as light intensity may also influence dustbathing behavior, as has been observed in red junglefowl, which delayed dustbathing until sufficient light and warmth were present (Hogan and Van Boxtel, 1993). To date, only Duncan et al. (1998) reported that hens prefer to dustbathe in bright light. However, this finding is likely confounded, as sunlight is associated with radiant heat. Therefore, this study aimed to investigate whether laying hens show a preference for a brighter or a darker environment when dustbathing by offering them a choice between four combinations, i.e., two dustbathing substrates (peat versus sand) and two light intensities (5 versus 100 lux). Multiple studies have established that peat and sand are preferred dustbathing substrates for chickens (Van Liere, 1991; Sanotra et al., 1995; De Jong et al., 2007). However, they differ in color and light reflectance. These differences may further influence substrate preferences for dustbathing, particularly in combination with light intensity. Therefore, it was decided to include both substrates in this study. During a preference test, the dustbathing frequency and duration in bright versus darker areas was recorded in order to assess the effects of substrate type, light intensity, and their interaction. In addition, visit frequency to the different choice compartments was recorded using RFID tracking. Based on the available literature, it was hypothesized that laying hens would prefer to dust bathe in peat under bright light conditions (De Jong et al., 2007), and would visit the bright compartments more frequently. The results of this study are expected to contribute to the establishment of ‘good practices’ for the design of dustbathing areas to facilitate laying hens in their need to dustbathe in a substrate and under a light intensity that matches their preference.

## Material and methods

The experimental procedures were reviewed and approved by the institutional Animal Welfare Body and classified as non-invasive in accordance with the Dutch Law on Animal Experiments (14 August 2025; registration number NAE_2025.W-025).

### Animals

The study took place in October 2025 with major experimental procedures described elsewhere (Broekmeulen et al., 2025). During the study, 24 white laying hens (Dekalb White, Hendrix Genetics, NL) were selected for behavioral testing. The hens were reared in a NivoVaria system (VDL Jansen, NL) with wood shavings and a light intensity of 5 lux. The hens arrived at our facilities at 17 weeks of age (WOA). Before testing, the hens were housed in pens with wood shavings (2.0 × 2.0 × 2.0 m). The barn lights (3000 K white light; flicker frequency: 2400 Hz) were set to 20 lux and a light period of 04:00 to 18:00 with a dimming period of 15 minutes. Upon arrival, each hen was fitted with a QR-coded backpack to enable individual identification during video observations. RFID tags were attached to the backpacks to track the hens’ visits to the different dustbathing compartments. It was assumed that the hens were habituated to the backpacks after the three weeks period prior to the start of behavioral testing at 20 WOA.

### Experimental design and preference testing

The study investigated whether laying hens prefer to dustbathe in a brighter or darker area using a preference test. During the test, hens could choose out of four different combinations of dustbathing substrates and light intensities: 1) sand 5 lux, 2) peat 5 lux, 3) sand 100 lux, and 4) peat 100 lux. During each round, four experimental pens were used simultaneously to test pairs of focal hens (n = 2/ preference pen). The study consisted out of three rounds, during which two new focal hens per pen were subjected to the preference test (n = 8/ round). Each round lasted seven days in total. The first round started when the hens were 20 WOA. The third and second round took place when the hens were 22 and 23 WOA, respectively. The first three days were used for habituation to the test environment, while the remaining four days were used for observations. Rounds concluded at 18:00 h on the seventh day of a round, when the pen lights switched off. The experimental pens consisted of a central compartment (2.0 × 1.0 m) and four dustbathing compartments (1.0 × 0.5 m). The central compartments were equipped with feeders, drinkers, perches and nests, and wood shavings were used as a litter substrate. The location of the dustbathing compartments was randomized per pen, to ensure that the location of the resources varied per preference pen. Hens gained access to the dustbathing compartments when stepping over a wooden barrier (height: 15 cm) in the compartment entrance (width: 0.5 m). In each pen, five LED lamps (ND-Dome, Once by Signify, NL) were installed: one lamp hung in the central compartment (20 lux; 3000 K white light; height: 95 cm) and four lamps were installed in the preference pen (height: 75 cm). Black plastic barriers were put up between the pens and compartments to prevent light pollution to other compartments. Light intensity was measured within each substrate and light compartment using a photometer (Sim-2 plus, MeTrue Inc., USA). Measurements were taken at chicken height (± 35 cm) at five locations: the center and the four corners of each compartment. An additional measurement was taken at floor level. In peat, the light intensity (mean ± SE) was measured at 6.57 ± 0.34 lux under 5 lux and 141.5 ± 13.3 lux under 100 lux. In sand, the light intensity was measured at 6.7 ± 0.2 lux under 5 lux and at 132.3 ± 7.4 lx under 100 lux. The spectral distribution of the lamps spanned 423.7–737.8 nm.

### Video observations and RFID registrations

Two IP cameras (HKVISION, CN; height: 3.30 m) were mounted above each dustbathing compartment to record continuous video during the test rounds. Video observations were conducted during the light period (i.e., 04:00–18:00) over four days per round, resulting in a total of 12 days of video observations. Videos were used to observe hens’ visits and dustbathing behavior and to link these to RFID data. For each visit, hen ID, visit frequency and duration relative to dustbathing, and the completeness of the dustbathing repertoire were manually annotated. A visit was recorded only if a hen stepped over the compartment barrier. Individual hens were identified via the QR-codes, which were visible on the video recordings due to the top view camera angle. A dustbathing repertoire was defined to start when a hen was observed tossing dust into her plumage and completed when she was observed shaking dust from her plumage (Van Liere, 1991).

In each dustbathing compartment, an RFID antenna (High Frequency, HF, DSLR1000, Freaquent Froschelectronics GmbH, AT; height: 40 cm) was installed to detect the hens’ RFID tags. The hens were tracked continuously from placement into the experimental pens up to the conclusion of the video observations. RFID registrations were recorded at 30 Hz with timestamps and antenna numbers, logged daily in CSV files. A visit was defined as a cluster of consecutive RFID registrations from the same individual, with intervals of less than 20 seconds between registrations at the same antenna. When hens were within range of the RFID antenna in the compartment, they were typically re-detected multiple times while present. An interval of more than 20 seconds was interpreted as the end of a visit and start of a new visit. RFID antennas were positioned above the entrances of the choice compartments and only detected hens when they were directly beneath the antenna. As a result, detections were intermittent rather than continuous, and hens could remain inside the compartment for extended periods without continuous detection. Due to this limitation, RFID data were used solely to quantify visit frequency and could not be linked to individual dustbathing events observed on video.

### Data analysis

Data were analyzed using R software (R version 4.5.1). Three statistical analyses were performed, with significance level at P < 0.05. Two analyses focused on the dustbathing observations, and one on the RFID tracking data. For the dustbathing dataset, only actual dustbathing events were included, analyzing dustbathing frequency and duration. In the RFID dataset, the visit frequency to each dustbathing compartment was analyzed. Complete and incomplete dust baths were combined due to the low number of observed dust baths (n = 59). The dustbathing frequency and visit frequency were analyzed using generalized linear mixed models (GLMMs) with a negative binomial distribution. Dustbathing duration was analyzed using a linear mixed model (LMM) after square-root transformation to meet the assumption of normally distributed residuals. In all models, the dustbathing substrate and light intensity combination was included as fixed effects, and hen ID as a random effect. Models were fitted using the R package `glmmTMB`, and pairwise comparisons were performed with the `emmeans` package. P-values were adjusted with Tukey’s method to correct for multiple comparisons.

## Results and discussion

**Fig. 1:**
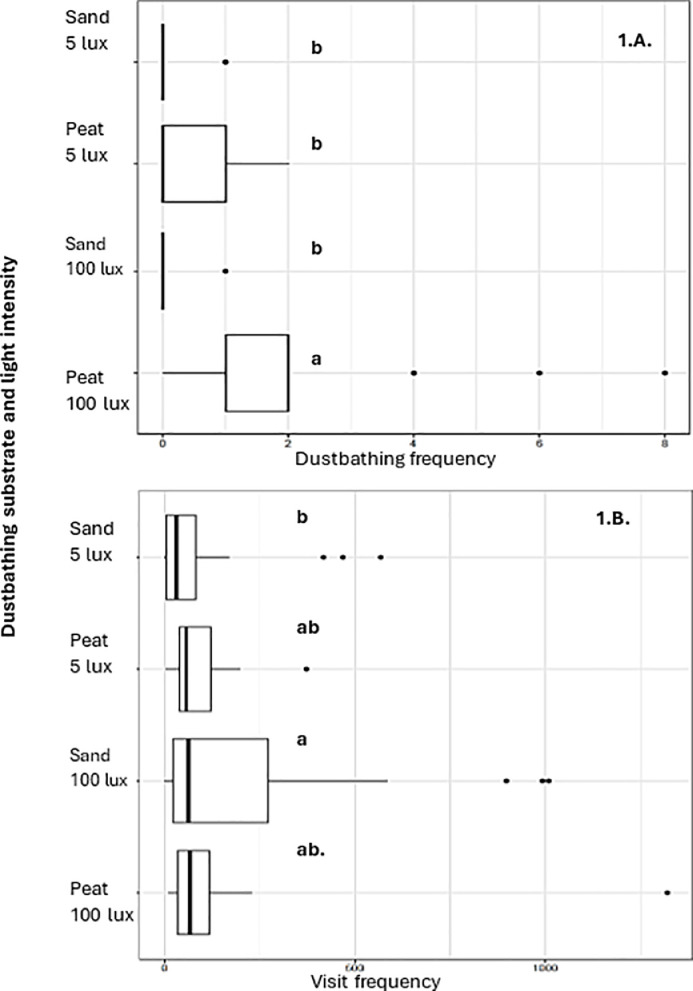
A. Boxplots of dustbathing frequency for each combination of substrate and light intensity. B. Boxplots of visit frequency for each combination of substrate and light intensity. Different letters indicate significant pairwise differences between compartments (P < 0.05). Compartments sharing the same letter do not differ significantly.

**Fig. 2:**
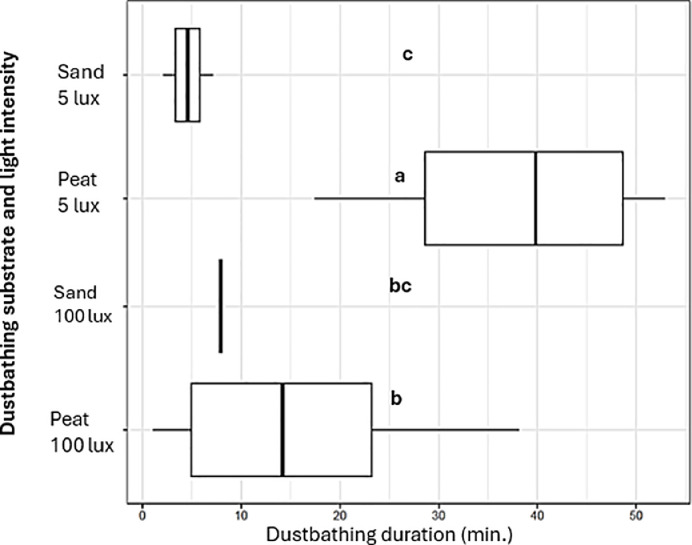
Boxplot of dustbathing duration for each substrate and light intensity. Different letters indicate significant pairwise differences between compartments (P < 0.05). Compartments sharing the same letter do not differ significantly.

Dustbathing frequency was affected by the combination of dustbathing substrate and light intensity (χ² = 43.23, df = 4, P < 0.001). Hens performed the majority of dust baths in compartments with peat at 100 lux (n = 43), which was 43.0 times more frequent than in sand at 100 lux (n = 1, SE = 44.7, P = 0.003), 5.4 times more than in peat at 5 lux (n = 8, SE = 2.4, P = 0.002), and 21.5 times more than in sand at 5 lux (n = 2, SE = 16.4, P < 0.001) (Fig. 1A). Hens also performed 5 dust baths in the central compartment with wood shavings at 20 lux. No differences in dustbathing frequency were detected between sand at 100 lux and peat at 5 lux (P = 0.3), sand at 100 lux and sand at 5 lux (P = 1.0), or peat at 5 lux and sand at 5 lux (P = 0.4). Most dust baths were completed, with 44 out of the 59 events following the full behavioral repertoire. Taken together, these findings show that the hens had a stronger preference to dustbathe in peat and 100 lux, which corresponds with the findings from De Jong et al. (2007) regarding substrate preference. During the present study, the hens could ‘easily’ access the dustbathing compartments by stepping over a barrier, whereas in the study by De Jong et al. (2007), hens had to push through weighted doors to gain access to dustbathing substrate, indicating that hens preferred peat for dustbathing, even when other substrates are accessible. The present study also showed that hens preferred to take a dust bath at a light intensity of 100 lux, which is consistent with our hypothesis. This finding may suggest preferences for substrate and brighter light may reinforce each other. To our knowledge, the present study is the first of its kind to examine which combination of substrate and light intensity is preferred by laying hens when dust bathing. Duncan et al. (1998) are the only ones to report that laying hens prefer to dustbathe in bright (sun)light. Interestingly, few dust baths were also taken in wood shavings at 20 lux, which might indicate that hens prefer a light intensity of 20 lux over 100 or 5 lux, given that wood shavings are generally not preferred as a dustbathing substrate compared to peat or sand (e.g., Van Liere, 1991; Sanotra et al., 1995; De Jong et al., 2007). The observed dustbathing in wood shavings might also be related to prior experience with this substrate, as wood shavings were also used as litter in the home pens (Van Liere, 1991; Sanotra et al., 1995). As only two hens were tested in each of the experimental pens, limited access or space to preferred substrates is an unlikely explanation. However, it cannot be excluded that a dominant hen performing dustbathing could have prevented the subordinate hen to dustbathe in the same area, resulting in dustbathing in wood shavings. The selected light intensities (5 and 100 lux) were chosen to create clear contrasts. However, preferred light intensity levels might lie between or outside these values, warranting further investigation. The visit frequency to the compartments, irrespective of dustbathing behavior, was influenced by the combination of dustbathing substrate and light intensity (χ ^2^ = 9.1072, df = 3, P = 0.028). In total, the laying hens visited the compartments 12,506 times. Compartments with sand at 100 lux were visited most frequently (n = 5,095), followed by peat at 100 lux (n = 3,135), sand at 5 lux (n = 2,226), and peat at 5 lux (n = 2,050). Hens visited compartments with sand at 100 lux more often than those with sand at 5 lux (5,095 vs. 2,226 visits; ratio = 2.5, SE = 0.8, P = 0.02; Fig. 1B). No differences in visit frequency were found between peat and sand at 100 lux (P = 0.8), peat at 100 lux and peat at 5 lux (P = 0.9), peat at 100 lux and sand at 5 lux (P = 0.2), sand at 100 lux and peat at 5 lux (P = 0.4), or sand at 5 lux and peat at 5 lux (P = 0.5). Overall, the visit frequency to the compartments largely exceeded the number of dustbathing events, indicating that hens also used the compartments for other activities, such as exploration and foraging. The higher visit frequency at 100 lux may reflect increased overall activity (Davis et al., 1999), with hens entering and leaving compartments more often even when not dustbathing. This pattern was observed in both substrates, although it was not statistically significant for peat. It is important to note that limitations of the RFID antenna set-up may have resulted in an overestimation of visit frequency, as hens could remain in the compartment without being detected. However, because this limitation applied equally across all compartments, it is unlikely to have affected the relative differences between substrates and light intensities. The duration of dust baths was influenced by the combination of dustbathing substrate and light intensity (χ ^2^ = 29.973, df = 4, P < 0.001). Across the 59 observed dust baths, durations ranged from 1:03 to 52:51 minutes. Complete dust baths lasted between 1:03 and 47:35 minutes, whereas incomplete dust baths ranged from 4:28 to 52:51 minutes. Video observations indicated that dustbathing events were concentrated around the middle of the light period, with 54% of events (32 out of 59) occurring between 09:30 and 12:30 (i.e., relative to a light period of 04:00-18:00). This pattern is consistent with previous observations (Hogan et al., 1993; Duncan et al., 1998; De Jong et al., 2007). Dust baths were longer in compartments with peat at 5 lux compared with peat at 100 lux (estimate = 21.98, SE = 5.72, P = 0.003), sand at 100 lux (estimate = 32.38, SE = 8.23, P = 0.002), and sand at 5 lux (estimate = 34.36, SE = 6.46, P < 0.001). In addition, dust baths in peat at 100 lux were longer than those in sand at 5 lux (estimate = 12.4, SE = 3.8, P = 0.02). No differences in dustbathing duration were found between peat at 100 lux and sand at 100 lux (P = 0.5), and sand at 100 lux and sand at 5 lux (P = 1.0; Fig. 2). Currently, it is unclear what the effects of the interaction between light intensity and dustbathing substrate is on the total duration of a dust bath, as both complete and incomplete dust baths varied largely in duration. The dustbathing duration is likely related to the effectiveness of a substrate in maintaining plumage condition. However, a limitation of this study is the limited number of observed dustbathing events and the large variation in duration, which has to be taken into consideration when interpreting the results.

While the compartments were assumed to allow two hens to dustbathe simultaneously, disturbances by more dominant pen mates or prior dust baths could have affected the duration. When designing functional areas in commercial poultry houses, sufficient space should be provided to prevent competition and to ensure that subordinate hens are not displaced by dominant hens (Leone and Estévez, 2008). Hens are often observed dustbathing in darker areas in commercial housing systems, which may reflect subordinate individuals avoiding disturbance from dominant pen mates when the motivation to dustbathe is high. This in combination with an intrinsic motivation to dustbathe could explain why dustbathing also occurs under suboptimal or non-preferred conditions. However, it remains unclear whether dustbathing under such conditions affects hen welfare. Grebey et al. (2020) showed that dustbathing in white laying hen hybrids is more strongly synchronized and of longer duration than in brown hybrids. Together with preferences for dustbathing substrate and light intensity combinations, this highlights important considerations for the design of functional dustbathing areas (Grebey et al., 2020). In conclusion, the current study demonstrates that laying hens show a clear preference for dustbathing in peat under brighter light. Our finding highlights the importance of both substrate and light intensity in dustbathing behavior. Social factors and space availability are likely to influence dustbathing behavior but merit further investigation. Moreover, our findings emphasize that in the design of functional dustbathing areas in commercial poultry houses should not only consider substrate but also light conditions to accommodate the hen’s intrinsic motivation to dustbathe. Future research should investigate a broader range of light conditions to better identify light preferences in laying hens for dustbathing. In addition, given concerns regarding environmental impact of peat moss, we recommend that future studies investigate alternative dustbathing substrates that meet the behavioral needs of hens while also being practical for on-farm application and having a lower environmental impact.

## CRediT authorship contribution statement

**Catharina M.H. Broekmeulen:** Writing – review & editing, Writing – original draft, Visualization, Data curation. **Thea G.C.M. van Niekerk:** Writing – review & editing, Methodology, Conceptualization. **Arjan van Dolderen:** Writing – review & editing, Investigation. **Rudi M. de Mol:** Writing – review & editing, Formal analysis, Data curation. **Dennis E. te Beest:** Writing – review & editing, Visualization, Formal analysis. **Ingrid C. de Jong:** Writing – review & editing, Validation, Supervision, Resources, Project administration, Methodology, Funding acquisition, Conceptualization.

## Disclosures

The authors declare that they have no known competing financial interests or personal relationships that could have appeared to influence the work reported in this paper.
